# Resignation of Prof. H. H. Childs

**Published:** 1863-05

**Authors:** 


### RESIGNATION OF PROF. H. H. CHILDS.

At a meeting of the Trustees of the Berkshire Medical College, held last Thursday, Hon. Henry H. Childs, the President of the Institution, as well as its founder and father, resigned the Professorship of "Obstetrics and the Diseases of Women and Children," which he has held so many years. Dr. Childs' advanced age rendered it necessary that he should seek some relief—although a hale and hearty old age is his, which we trust will enable him to hold for years the Presidency of the College, which he still retains. In accepting the resignation the Board adopted unanimously the following resolutions:

*Resolved*, That the resignation of Dr. Childs requires from us more than a passing notice. For nearly forty years he has been the active head of the Berkshire Medical College—his usefulness having extended to a period almost unprecedented. During these years, by his energy, zeal and enthusiasm, he has achieved a wide-spread reputation as a medical man, and by his kindness of heart, and courtesy of manner, a no less deserved name as a Christian gentleman. He has ever maintained a high standard of medical honor, and his pupils must forget or ignore his teachings before they could stoop to anything ignoble or ungenerous. With a quick appreciation of merit, however modest, and ever ready with the kindly word of needed encouragement, his pupils learned to love him, and thousands, through the length and breadth of our land, affectionately look back to him as a kindly foster-father.

While we regret the infirmities which compel the retirement of our venerable President, as an active instructor, we earnestly hope that his interest in the institution, which is so identified with his life and name, may not abate, and that he may long be spared to speak words of cheer to the new generation of students, and to give the benefit of his advice and counsel to the Faculty and Trustees.

